# Typical Presentation to Rare Diagnosis: A Case Report of Appendicitis Revealing Cecal Adenocarcinoma

**DOI:** 10.7759/cureus.66956

**Published:** 2024-08-15

**Authors:** David A Eppley, Angelica R Carnemolla, Steven Perez-Bello, Lisett Castellanos, Carlos J Bello

**Affiliations:** 1 College of Osteopathic Medicine, Lake Erie College of Osteopathic Medicine, Bradenton, USA; 2 Biology, Florida International University, Miami, USA; 3 Pediatrics, Larkin Community Hospital South Miami, South Miami, USA; 4 General Surgery, Larkin Community Hospital Palm Springs, Hialeah, USA

**Keywords:** malignant tumor resection, appendicitis in elderly, right-sided hemicolectomy, atypical appendicitis, cecal adenocarcinoma

## Abstract

Appendicitis is predominantly observed in teens and young adults. While typical causes include fecalith-induced luminal obstructions, in older adults less common etiologies such as tumors should be considered. This report highlights a rare case of appendicitis secondary to cecal adenocarcinoma in a high-risk patient with a history of obesity and type 2 diabetes mellitus. This case underscores the necessity of considering malignancy as a differential diagnosis in older adults presenting with appendicitis-like symptoms.

## Introduction

Appendicitis is most common in teens and young adults with a lifetime risk of approximately 7%, and a higher prevalence in males [1]. Clinically, these patients typically present with periumbilical pain that localizes to the right lower quadrant, along with nausea, vomiting, and a mild fever [1,2]. However, the incidence of appendicitis and its etiologies vary with age. In individuals over 50, appendicitis becomes less common and the causes often shift from the typical pathology to less common factors [1]. Typical pathology consists of a fecalith causing luminal obstructions which account for 50-80% of cases in the common younger age groups [1]. Less common factors such as gallstones, parasitic masses, and most notably tumors, can be an atypical cause of appendicitis, particularly in older adults [1].

Tumors of the appendix are a critical consideration. Neuroendocrine tumors (NETs) or carcinoids are the most common appendiceal tumors and are usually small and incidentally found [1,3]. However, adenocarcinomas, which are typically larger, are more likely to cause clinical or lab findings such as fatigue and weakness due to iron deficiency anemia [1]. More rarely, large adenocarcinomas can cause clinical findings, such as acute appendicitis, by mimicking its symptoms due to the mass effect causing luminal obstruction [1]. To highlight the rarity, 8-10% of colon cancers occur in the cecum, with a small fraction of cecal cancer progressing to a clinical level where it may cause appendicitis [4].

Certain relationships become apparent when comparing risk factors in patients with and without neoplastic disease. Neoplasms, like colon adenocarcinoma, are more common in men, individuals over 50, and those with gastrointestinal diseases that predispose to cancer [5]. Obesity, low physical activity, smoking, and high consumption of salt and red meat are factors that increase colorectal cancer risk [1,5].

## Case presentation

A 55-year-old Hispanic female with a past medical history of obesity and type 2 diabetes mellitus presented to the emergency room reporting intermittent abdominal pain located in the epigastrium and right lower quadrant that started two weeks ago. The patient reported that this was the first time she experienced the pain, which she described as an 8/10 intensity that radiated to the lower abdomen.

**Figure 1:**
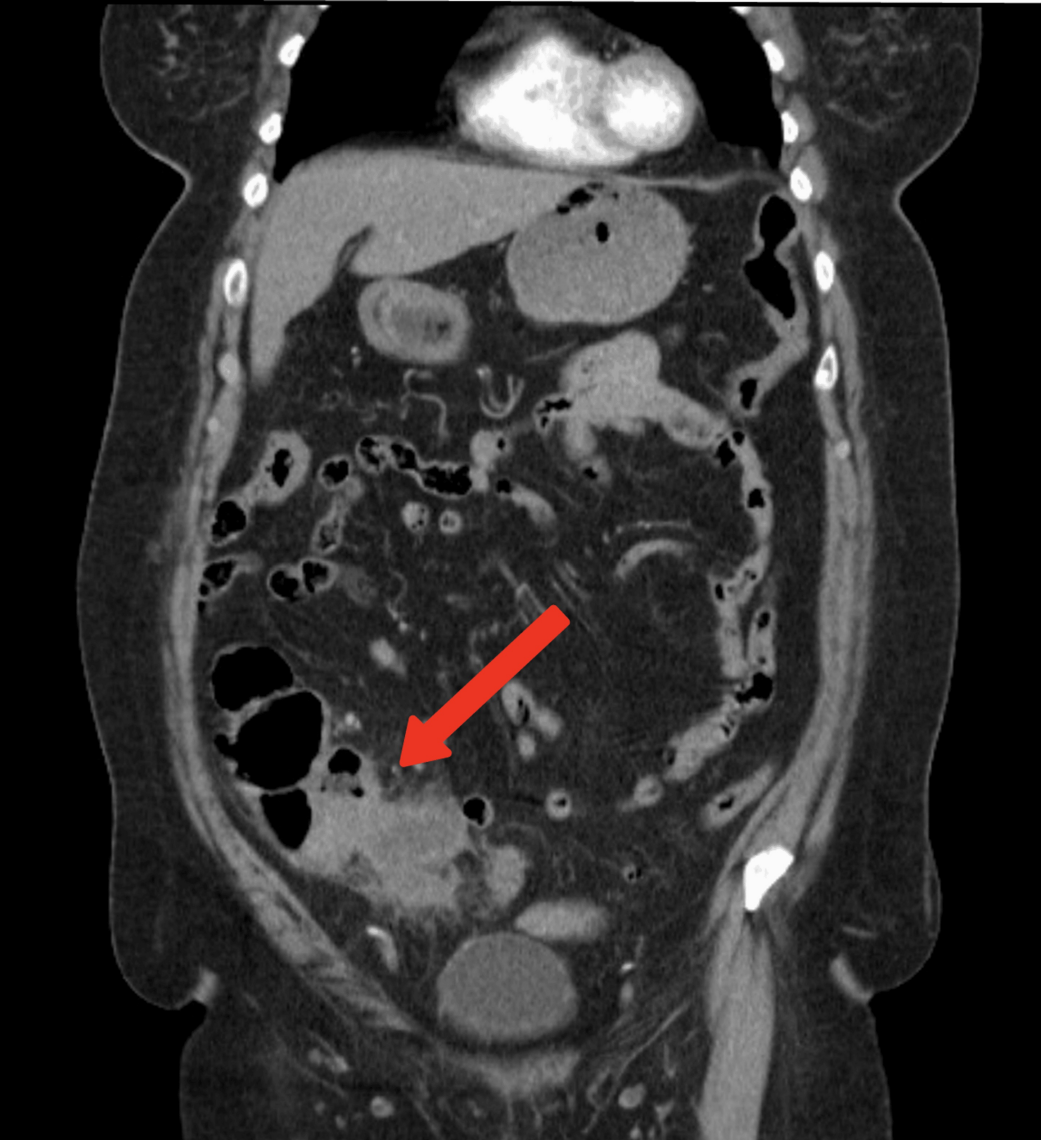
CT scan of abdomen and pelvis - coronal view

The patient had stable vital signs. A physical exam displayed a tender abdomen in the right lower quadrant with rebound tenderness located over McBurney’s point. A CT scan of the abdomen and pelvis showed an enlarged appendix up to 1.4 cm with significant surrounding fat stranding (Figure 1). Further description of the appendix showed hypodense fluid collection, measuring 3 x 3.4 x 2.6 cm, with rim enhancement abutting the terminal ileum. Labs were significant for a complete blood count showing a neutrophil-predominant shift with leukocytes within a normal range. 

Considering the patient showed a clinical presentation consistent with acute appendicitis, this became the most likely diagnosis with localized peritonitis. Consent for laparoscopic appendectomy was obtained.

The laparoscopic procedure was initiated with no complications. Upon further inspection towards the right lower quadrant, a large inflammatory mass involving the cecum, terminal ileum, and redundant sigmoid colon was found. The examination was positive for a large cecal mass that extended to the serosa and lateral abdominal wall. Post-operative differential diagnosis aimed to distinguish between phlegmon versus carcinoma.

**Figure 2:**
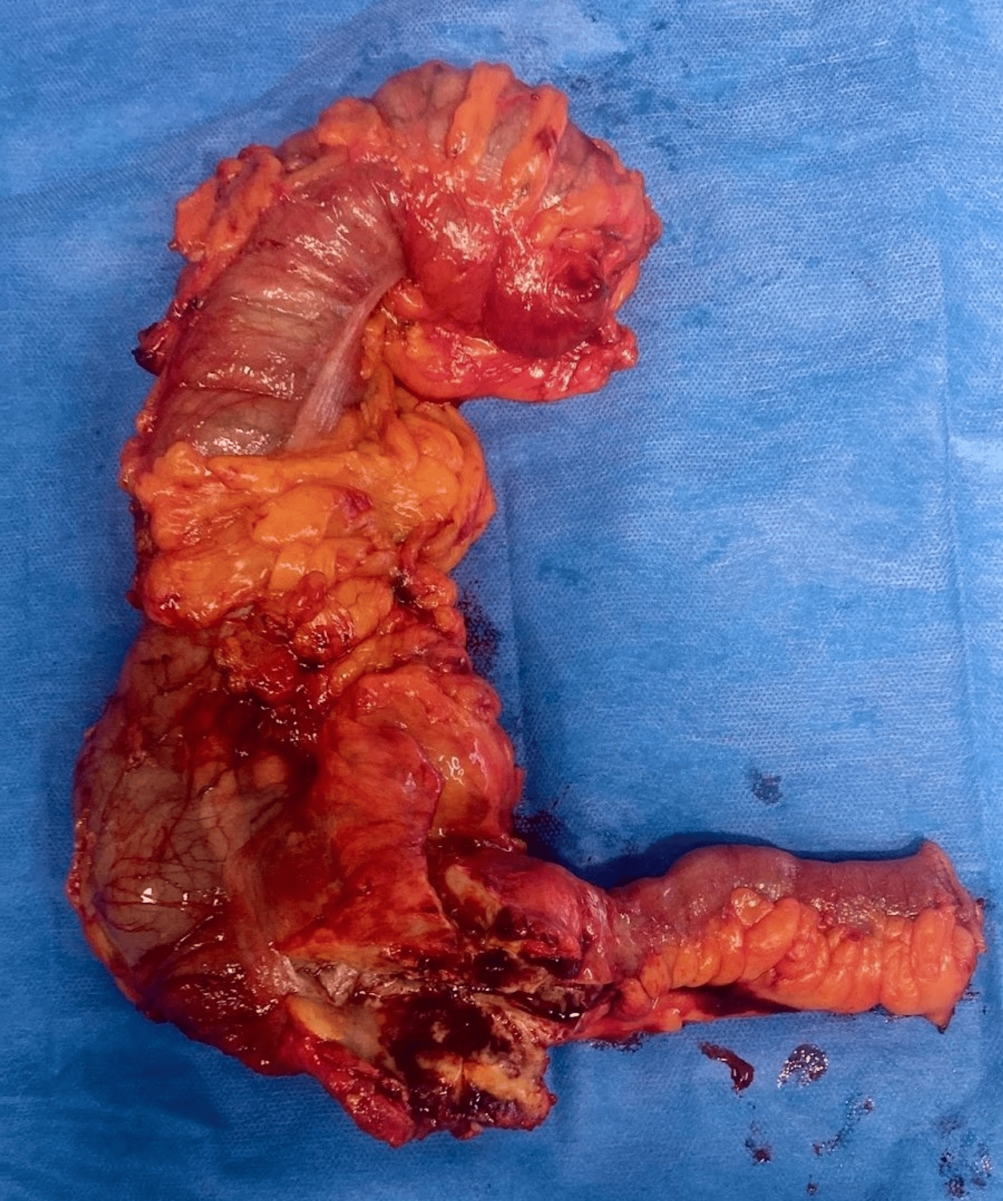
Resected specimen

The decision was made to perform a right hemicolectomy. The terminal ileum, approximately 15 cm proximal to the ileocecal valve, was transected as well as the mid-transverse colon (Figure 2). The umbilical incision made intuitively for the laparoscopy was extended another 3.5 inches to allow removal of the mass. The specimen was a large mass with several large lymph nodes attached to it. After stabilization, the patient was discharged from the hospital with a referral to gastroenterology for a post-operative colonoscopy to be performed 6-8 weeks later.

**Figure 3:**
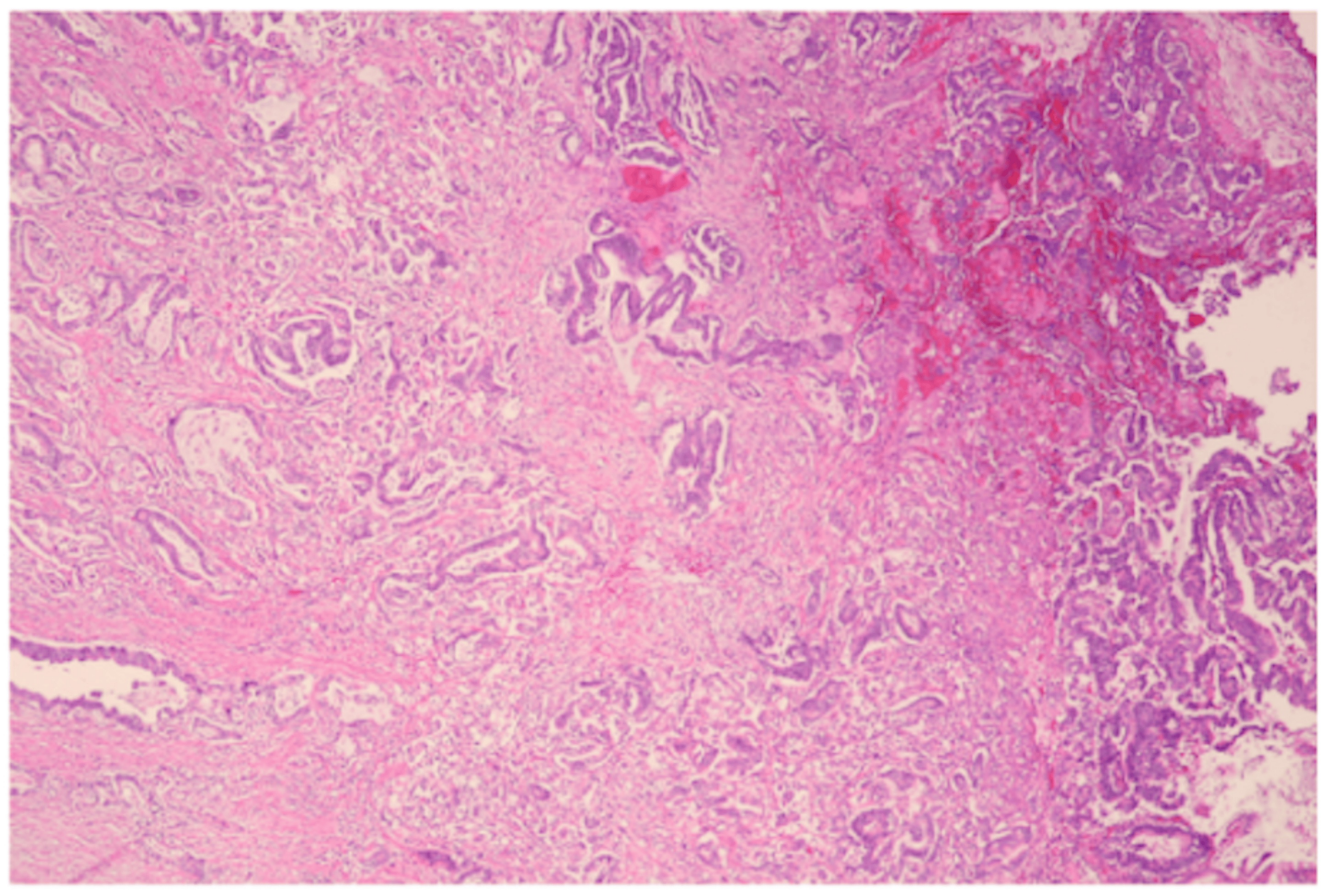
Histological specimen

**Figure 4:**
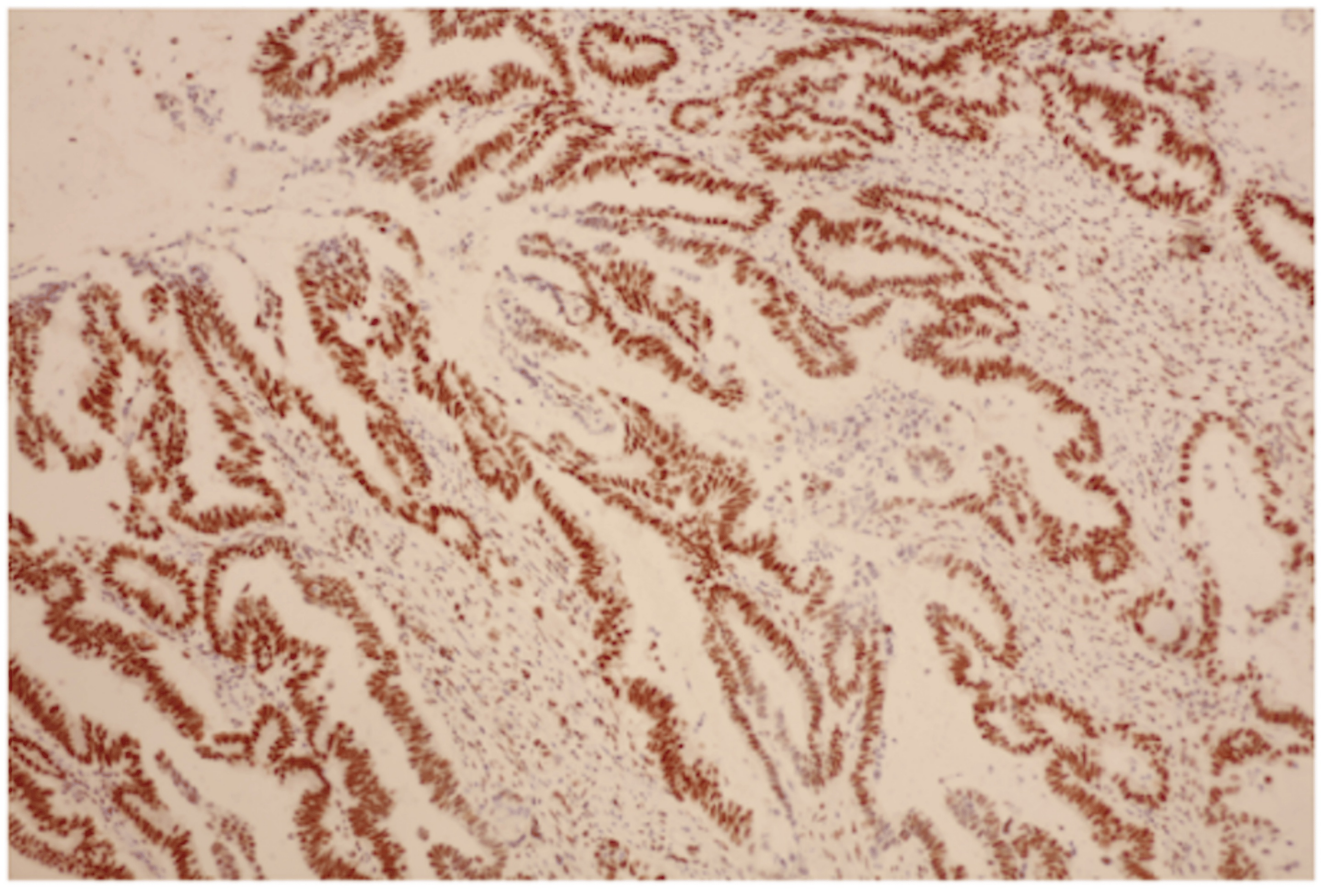
MLH1 immunohistochemistry staining

The pathology report indicated that the tumor was an adenocarcinoma, with the greatest dimension of 2.5 cm. A histological specimen was obtained (Figure 3). It was classified as moderately differentiated (G2). Out of 13 regional lymph nodes examined, one was found to be involved. The pathologic stage classification written in the pathology report, according to the American Joint Committee on Cancer (AJCC) Eighth Edition, was pT3. This indicated that the tumor invaded through the muscularis propria into the subserosa or mesoappendix [6]. It was also classified as pN1, indicating one to three regional lymph nodes were positive for tumor cells [6]. Additionally, there was acute typhlitis with fistulous tracts and abscess formation, as well as a sessile serrated lesion in the distal appendix. No distant metastasis was confirmed pathologically in this case. Microsatellite instability was seen with MLH1 staining (Figure 4). Immunohistochemistry results were significant for antibody classes MLH1, MSH2, and PMS2 (Table 1). Given these pathology report findings, follow-up with oncology was recommended.

**Table 1:** Immunohistochemistry results

Antibody Name (Clone)	Result	Positive Cells (%)
MLH-1 (G168-15)	Positive	95
MSH2 (FE11)	Positive	90
MSH6 (BC-44)	Positive	40
PMS2 (A16-4)	Positive	95

## Discussion

In the context of appendiceal diseases, particularly in high-risk patients as defined earlier, it is critical to consider adenocarcinoma as a differential when a patient presents with appendiceal-like clinical symptoms. The incidence and causes of appendicitis in the elderly differ significantly from young typical populations. A study that reviewed 218 patients, aged 65-95, found that only 1.8% of appendicitis cases were due to carcinoma of the cecum [2]. Regardless of the exact percentage, adenocarcinoma-inducing appendicitis is a rare, yet, potential cause that should be considered.

Delayed presentation of appendicitis in adults and the elderly increases the risk of perforation, emphasizing the need for prompt surgical intervention to prevent complications [7]. Management of appendiceal masses, which can be inflammatory or malignant, poses a significant challenge. Due to the uncertainty of the mass being benign or malignant, right hemicolectomy is often recommended to prevent the potential spread of malignancy if the mass perforates [4,7,8]. This approach was highlighted by a study where 48 patients out of 3032 presented with masses suspicious of malignancy and four were confirmed to have malignant neoplasms post-hemicolectomy [7]. 

In the current case, an immediate right hemicolectomy was deemed appropriate due to the involvement of a large section of the colon and the uncertainty of the mass's nature. While some studies have advised confirming malignancy before proceeding with extensive surgery, the risks associated with delayed intervention, particularly in older adults prone to perforation, warranted this decision [4]. Although a less invasive procedure, such as an ileocecal resection, may be more favorable due to lower operative time, lower morbidity rate, and shorter post-operative hospital stay in cases with less extensive involvement, this more conservative approach was not deemed appropriate in this case [8]. Post-operative recommendations for patients over 40 with appendicitis should include a colonoscopy to rule out mass-induced appendicitis, considering the higher likelihood of malignancy in this age group [4]. Therefore, it was recommended for the patient to follow-up with gastroenterology after discharge.

The pathology findings further support the need for vigilance. It is vital to consider family history in older adults, especially in high-risk populations such as those with hereditary nonpolyposis colorectal cancer (HNPCC) [9]. In this case, it would be favorable to explore the patient’s family history of malignancies such as endometrial and colorectal to consider HNPCC as a cause of the patient’s microsatellite instability rather than spontaneous mutations [10]. This would allow for appropriate screening of relatives. This case underscores the importance of maintaining a high index of suspicion for malignancy in older adults presenting with appendiceal symptoms in higher-risk patients such as those who are overweight, smokers, sedentary, or who are revealed to have genetic or acquired mutations [5]. Clinicians should maintain a more open differential diagnosis of the underlying cause of appendicitis, even when the clinical picture appears clear-cut.

## Conclusions

This case illustrates the critical importance of considering cecal adenocarcinoma as a differential diagnosis in older adults presenting with symptoms of appendicitis. The patient's clinical presentation, risk factors, and intraoperative findings necessitated a right hemicolectomy to address the malignancy and prevent its spread. This report emphasizes the need for comprehensive evaluation and timely surgical intervention in managing appendicitis in high-risk populations. Vigilance and thorough investigation are essential to ensure appropriate diagnosis and treatment, highlighting the broader implications for clinical practice in similar presentations.
